# The cytotoxic molecule granulysin is capable of inducing either chemotaxis or fugetaxis in dendritic cells depending on maturation: a role for V*δ*2^+^ *γδ* T cells in the modulation of immune response to tumour?

**DOI:** 10.1111/imm.13248

**Published:** 2020-09-16

**Authors:** Emma L. Sparrow, Daniel W. Fowler, Joe Fenn, Jonathan Caron, John Copier, Angus G. Dalgleish, Mark D. Bodman‐Smith

**Affiliations:** ^1^ Infection and Immunity Research Institute St. George’s University of London SW17 0RE London UK; ^2^ Antibody and Vaccine Group Centre of Cancer Immunotherapy Southampton General Hospital University of Southampton Southampton UK

**Keywords:** chemotaxis, dendritic cells, granulysin, *γδ* T cells

## Abstract

Release of granulysin by *γδ* T cells contributes to tumour cell killing. A cytolytic 9000 MW isoform of granulysin kills tumour cells directly, whereas a 15 000 MW precursor has been hypothesized to cause both the maturation and migration of dendritic cell (DC) populations. Recruiting DC to a tumour is beneficial as these cells initiate adaptive immune responses, which contribute to the eradication of malignancies. In this study, V*δ*2^+^ *γδ* T cells were activated by stimulation of peripheral blood mononuclear cells with zoledronic acid or Bacillus Calmette–Guérin (BCG), or were isolated and cultured with tumour targets. Although a large proportion of resting V*δ*2^+^ *γδ* T cells expressed 15 000 MW granulysin, 9000 MW granulysin expression was induced only after stimulation with BCG. Increased levels of activation and granulysin secretion were also observed when V*δ*2^+^ *γδ* T cells were cultured with the human B‐cell lymphoma line Daudi. High concentrations of recombinant 15 000 MW granulysin caused migration and maturation of immature DC, and also initiated fugetaxis in mature DC. Conversely, low concentrations of recombinant 15 000 MW granulysin resulted in migration of mature DC, but not immature DC. Our data therefore support the hypothesis that V*δ*2^+^ *γδ* T cells can release granulysin, which may modulate recruitment of DC, initiating adaptive immune responses.

## Graphical abstract

V*δ*2^+^ *γδ* T cells are capable of the release of two isoforms of granulysin; a cytolytic 9000 MW isoform, which kills tumour cells directly, and a 15 000 MW precursor, which has been hypothesized to cause both the maturation and migration of dendritic cell (DC) populations. Recruiting DC to a tumour is beneficial as these cells initiate adaptive immune responses, contributing to the eradication of malignancies. In this study, we show that high concentrations of recombinant 15 000 MW granulysin cause migration and maturation of immature DC, and can also initiate fugetaxis in mature DC, supporting the hypothesis that granulysin released by V*δ*2^+^ *γδ* T cells may modulate recruitment of DC, influencing initiation of adaptive immune responses.

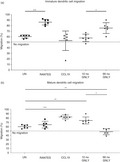

**Abbreviations**
BCG: Bacillus Calmette–Guérin
DC: dendritic cell
FSC: forward scatter
HLA‐DR: human leucocyte antigen‐D‐related
HMBPP: (E)‐4‐hydroxy‐3‐methyl‐but‐2‐enyl pyrophosphate
IPP: isopentenyl pyrophosphate
MACS: magnetic activated cell sorting
PBMC: peripheral blood mononuclear cells
SDF‐1: stromal cell derived factor 1
SSC: side scatter
ZA: zoledronic acid

## Introduction

A small subset of T cells possess a T‐cell receptor composed of *γ* and *δ* chains rather than *α* and *β* chains, and these *γδ* T cells account for up to 5% of the T cells found within human peripheral blood.^1^ Although the proportion of *γδ* T cells in the T‐cell population as a whole is low, this subset does not require processing and presentation of antigen to become activated, allowing a rapid response to infected or malignant target cells.

Previous research has shown evidence that *γδ* T cells bearing a V*δ*2 chain, comprising approximately 80% of the *γδ* T‐cell population found in the peripheral blood of humans,^2^ are capable of recognizing phosphoantigens such as prenyl pyrophosphates. These are intermediates of the isoprenoid synthesis pathways, present within both bacteria and eukaryotes. Within bacteria, the phosphoantigen (E)‐4‐hydroxy‐3‐methyl‐but‐2‐enyl pyrophosphate (HMBPP) is produced in the 2‐C‐methyl‐d‐erythritol‐4‐phosphate pathway, and its eukaryotic homologue isopentenyl pyrophosphate (IPP) is produced in the mevalonate pathway.^3^ Research has shown that V*δ*2^+^ *γδ* T cells are activated by cells that accumulate HMBPP and/or IPP.^4^ Although the exact mechanism by which these cells recognize phosphoantigens remains to be fully elucidated, the current hypothesis suggests that intracellular binding of phosphoantigens to the molecule butyrophilin 3A1 is involved.^5^, ^6^, ^7^ HMBPP has been found to be substantially more stimulatory than IPP to V*δ*2^+^ *γδ* T cells, allowing these cells to easily differentiate foreign bacteria from self cells.^8^ Although the level of IPP within healthy eukaryotic cells is not usually sufficient to cause activation of V*δ*2^+^ *γδ* T cells, this molecule is overexpressed in some tumours in which the mevalonate pathway is dysregulated.^9^ Additionally, nitrogen‐containing bisphosphonate drugs such as zoledronic acid (ZA) can artificially elevate the level of IPP within cells, because of their inhibition of enzymes involved in the mevalonate pathway, resulting in an accumulation of IPP within the cell.^10^

Granulysin is a cytotoxic effector molecule, used by several immune cell populations to kill pathogens, in addition to infected or transformed cells. *γδ* T‐cell expression of this molecule has been shown to be pivotal in the immune response to both *Mycobacterium tuberculosis* and *Plasmodium falciparum*, as well as in several types of tumour.^11^, ^12^, ^13^ The 9000 MW isoform of granulysin has been shown to be directly cytotoxic, and co‐localizes with other cytotoxic molecules such as granzymes.^14^, ^15^ Evidence suggests that the 15 000 MW full‐length isoform, initially thought of as an inert precursor, could also have distinct immune functions. The 15 000 MW granulysin localizes to lysosome‐related effector vesicles,^15^ and has been shown to cause the maturation of immature DC populations, and the migration of both immature and mature DC, in addition to monocytes, memory *αβ* T cells and natural killer (NK) cells.^16^, ^17^, ^18^, ^19^

In this paper, we show that V*δ*2^+^ *γδ* T cells are capable of secreting granulysin in response to tumour. In addition, we show that recombinant 15 000 MW granulysin can cause the migration and maturation of DC, and propose that 15 000 MW granulysin has a dual migratory function; we found that immature DC migrate towards high concentrations of 15 000 MW granulysin, whereas mature DC migrated only towards low concentrations of this molecule, and in fact migrated away from higher concentrations of 15 000 MW granulysin. This suggests the ability of 15 000 MW granulysin to induce both chemotaxis and fugetaxis of DC, in a concentration‐dependent manner and depending on DC maturation status. We therefore propose that the degranulation of V*δ*2^+^ *γδ* T cells in response to tumour can recruit and mature DC, leading to the initiation of an adaptive immune response to tumour antigens.

## Materials and methods

#### Peripheral blood mononuclear cell isolation

Whole blood samples taken from healthy donors were sourced from anonymized leucocyte blood cones supplied by the UK Blood Transfusion Service, London, UK or were collected from consenting healthy volunteers at St George’s, University of London, Tooting, UK. Peripheral blood mononuclear cells (PBMC) were isolated from whole blood samples by density‐adjusted centrifugation using Histopaque 1077 (Sigma‐Aldrich, Dorset, UK). Residual red blood cells were removed through addition of ammonium–chloride–potassium lysing buffer (Thermo Fisher Scientific, Waltham, MA), and contaminating platelets were eliminated by three slow‐speed centrifugations (200 ***g***, 10 min), in RPMI‐1640 medium (Sigma‐Aldrich). Finally, PBMC were resuspended in freezing medium [composed of 45% (volume/volume; v/v) RPMI‐1640 medium, 45% (v/v) fetal buffered saline and 10% (v/v) dimethylsulphoxide; all Sigma‐Aldrich] and initially frozen at −80°, before being transferred to liquid nitrogen for extended storage.

#### Cell isolation

CD14^+^ monocytes and *γδ* T cells were isolated from PBMC using magnetic activated cell sorting (MACS). In order to isolate *γδ* T cells, non‐*γδ* T cells (*αβ* T cells, NK cells, monocytes, B cells, DC, stem cells, granulocytes and erythroid cells) were depleted from PBMC using a *γ*/*δ* ^+^ T Cell Isolation kit from Miltenyi Biotec (Bergisch Gladbach, Germany). Monocytes were isolated through positive selection of CD14^+^ cells using CD14 microbeads from Miltenyi Biotec. The purity of each isolated cell population was assessed by flow cytometry, and was >90% for *γδ* T‐cell populations and >95% for monocyte populations.

#### Cell cultures

All cells were cultured in RPMI‐1640 medium (Sigma‐Aldrich) supplemented with 10% (v/v) fetal buffered saline (Sigma‐Aldrich), 10 000 U/ml penicillin and 10 000 µg/ml streptomycin (Thermo Fisher Scientific).

For experiments involving PBMC, 1 × 10^6^ cells were seeded in 96‐well round‐bottomed tissue‐culture plates (Corning, Corning, NY) in a total volume of 200 μl supplemented RPMI‐1640 medium. The following reagents were used to stimulate cells as required: 10 μg/ml BCG (Danish strain 1331; Statens Serum Institut, Copenhagen, Denmark), 5 μm ZA, 30 ng/ml phorbol 12‐myristate 13‐acetate (PMA), and 1 μg/ml ionomycin (all from Sigma‐Aldrich).

For *γδ* T‐cell expansion experiments, 5 × 10^5^ PBMC were seeded in a total volume of 200 μl supplemented RPMI‐1640 medium in 96‐well round‐bottomed tissue‐culture plates. Zoledronic acid (5 µm; Sigma‐Aldrich) and 15 ng/ml (315 U/ml) interleukin‐2 (IL‐2) (R&D Systems, Minneapolis, MN) were added to the medium. Cells were then cultured for 9 days before isolation of the *γδ* T‐cell population by MACS, and fresh supplemented RPMI‐1640 medium and IL‐2 were added every 2–3 days.

Daudi and Raji B‐cell lymphoma lines (European Collection of Authenticated Cell Cultures, Salisbury, UK) were used in experiments as *γδ* T‐cell‐susceptible and ‐resistant target cells, respectively. Tumour cells were cultured in 75‐cm^2^ tissue‐culture flasks at a recommended density of 4 × 10^5^ cells/ml in supplemented RPMI‐1640 medium (Sigma‐Aldrich) and were passaged every 2–3 days to maintain the recommended cell density.

For co‐culture experiments, 5 × 10^5^ expanded and isolated *γδ* T cells/ml and 5 × 10^5^ tumour cells/ml were added to a total volume of 200 μl supplemented RPMI‐1640 medium in 96‐well round‐bottomed tissue‐culture plates, at a 1 : 1 ratio of target to effector cells. Cells were cultured for 24, 48 or 72 hr before being harvested.

#### DC differentiation and maturation

Isolated peripheral blood CD14^+^ monocytes were seeded into six‐well flat‐bottomed tissue‐culture plates at a density of 1 × 10^6^ cells/ml in a total volume of 3 ml supplemented RPMI‐1640 medium. Then, 100 ng/ml (2900 U/ml) IL‐4 and 50 ng/ml (750 U/ml) granulocyte–macrophage colony‐stimulating factor (GM‐CSF (both R&D Systems)) were added to the medium, and cells were cultured for 7 days. Half of the total volume of medium was replaced every 2–3 days with fresh medium containing 100 ng/ml (2900 U/ml) IL‐4 and 50 ng/ml (750 U/ml) granulocyte–macrophage colony‐stimulating factor. Following 7 days of culture, light microscopy and flow cytometry were used to confirm the differentiation of CD14^+^ monocyte populations into immature DC. To test maturation, immature DC were treated for 24 hr with 100 ng/ml recombinant lipopolysaccharide (LPS) or 66 nm recombinant 15 000 MW granulysin (both R&D Systems). The purity of recombinant 15 000 MW granulysin used was determined by the manufacturer to be >95% by sodium dodecyl sulphate–polyacrylamide gel electrophoresis, and endotoxin contamination was assessed to be <1·0 EU per 1 μg of the protein by the limulus amoebocyte lysate method.

#### Flow cytometry

Cells were washed in flow cytometry buffer [phosphate‐buffered saline supplemented with 1% (weight (w)/v) bovine serum albumin, 0·1% (w/v) sodium azide and 0·5 mm EDTA (all Sigma‐Aldrich), and stained with fluorochrome‐conjugated antibodies according to the manufacturer’s instructions. Fc receptor blocking solution was added to flow cytometry buffer at a ratio of 1 : 20 before staining, to prevent non‐specific binding (BioLegend, San Diego, CA). Following staining, cells were washed three times in flow cytometry buffer, before being fixed with 4% (w/v) paraformaldehyde (BD Biosciences, Oxford, UK). For experiments involving intracellular staining, 3·5 μm brefeldin A (Sigma‐Aldrich) was added for the final 3 hr of culture to block protein trafficking. Following any required surface staining, cells were simultaneously fixed and permeabilized using 4% (w/v) paraformaldehyde and 0·1% (v/v) saponin (Cytofix/Cytoperm kit; BD Biosciences) before staining with fluorochrome‐conjugated antibodies, according to the manufacturer’s instructions. The following antibodies were used: Alexafluor 488–15 000+ 9 000 MW granulysin(RB1), phycoerythrin (PE)‐CD56(B159), fluorescein isothiocyanate (FITC)‐CD8(RPA‐T8) (all BD Biosciences), Alexafluor 647–9 000 MW granulysin(DH2), PE‐dazzle‐CCR5(J418F1), FITC‐CCR7(G043H7), FITC‐CD107a(H4A3), FITC/allophycocyanin (APC)‐CD27(M‐T271), Peridinin chlorophyll protein (PerCP)‐CD3(OKT3), FITC/APC‐CD45RA(HI100), FITC/APC‐CD69(FN50), PerCP‐Cy5.5‐CD80(2D‐10), Alexafluor 647‐human leucocyte antigen‐D‐related (HLA‐DR) (L243) (all BioLegend), PE‐CD14(TÜK4), APC‐Granzyme B(REA226), PE‐V*δ*1(REA173), PE‐V*δ*2(REA771), PE‐*γδ* T‐cell receptor (11F2) (all Miltenyi Biotec). For all experiments, matched isotype controls were used to determine levels of non‐specific binding.

To measure degranulation, anti‐CD107a antibodies and 1 μm monensin (Sigma‐Aldrich) were added for the final 4 hr of culture, before harvesting cells for staining. To measure tumour cell death, a live/dead discrimination dye (Thermo Fisher Scientific) was used, allowing quantification of dead Raji or Daudi cells by flow cytometry. The dye was diluted 100‐fold in FACS buffer containing cells to be stained. The cells were then incubated for 30 min at room temperature before being stained with a FITC‐conjugated antibody specific for CD19, allowing identification of tumour cells. Peaks of fluorescence representing live and dead cells were established before commencement of experiments using viable and heat‐killed tumour cells, respectively, and tumour cell death calculated as the percentage fluorescence observed within each condition, which was above that previously established to represent live cells.

Stained cells were run on an LSRII flow cytometer (BD Biosciences), and data were analysed using facsdiva (BD Biosciences) or flowjo (FlowJo LLC, Ashland, OR) software.

#### ELISA

The concentrations of granulysin, granzyme B and interferon‐*γ* (IFN‐*γ*) that were present within co‐culture supernatants were determined by ELISA (R&D Systems). The commercial granulysin ELISA could not distinguish between the 15 000 and 9000 MW isoforms of granulysin, and as such, data reflect the concentration of total granulysin only. Briefly, plates were coated with a mouse anti‐human antibody specific for each protein of interest. A twofold, seven‐point serial dilution of each protein was performed to generate a standard curve, and samples of unknown concentration were added to the plate. Biotinylated mouse anti‐human antibodies specific for each protein were added, followed by streptavidin–horseradish peroxidase. Finally, a 1 : 1 solution of hydrogen peroxide and 3,3′,5,5′‐tetramethylbenzidine was added to plates to induce a colour change, and the reaction was stopped by addition of 2 m sulphuric acid. Between each step, plates were washed three times in wash buffer (0·05% Tween‐20 diluted in phosphate‐buffered saline; Sigma‐Aldrich). The final absorbance of each sample was read at 450 nm and protein concentrations were interpolated from the standard curve using a four‐parameter logistic model provided by graphpad prism.

#### Ibidi μ‐migration assays

Migration of DC populations was assessed using Ibidi μ‐migration assays, performed according to the manufacturer’s instructions (Ibidi, Martinsried, Germany). In brief, immature or mature DC were diluted to a concentration of 3 × 10^6^ cells/ml in collagen gel, and added to the cell chamber of a μ‐migration slide (Ibidi). Unsupplemented medium was added to each chemoattractant chamber of the μ‐migration slide, and chemoattractants of interest (500 ng/ml RANTES, 2 ng/ml CCL19, 10 or 66 nm recombinant 15 000 MW granulysin; all R&D Systems) were added to one chemoattractant chamber to produce a concentration gradient across the slide. Migration was monitored using a time‐lapse microscope (Olympus IX70 inverted system; Olympus Corporation, Tokyo, Japan) equipped with a Hamamatsu C4742‐95 digital camera and a motorized stage controlled by image pro‐plus software (Media Cybernetics, Rockwell, MD, USA). The microscope and stage were enclosed within a heated (37°) humidified chamber (Solent Scientific, Portsmouth, UK) at 5% CO_2_. Images were captured every 15 min over a period of 24 hr and were then used to analyse the migration of cells towards each chemoattractant, using imagej software (National Institutes of Health, Bethesda, MD).

#### Statistical analyses

All statistical analyses were carried out using graphpad prism software (Prism 7; GraphPad Software, San Diego, CA). Significance was determined using either one‐way or two‐way analysis of variance or paired *t*‐tests, assuming Gaussian distributions in all cases. Unless otherwise stated, data are presented as mean ± standard deviation (SD). Statistical differences with *P*‐values <0·05 are reported in the figures (*, **, *** and **** are used to report *P*‐values of <0·05, <0·01, <0·001 and <0·0001, respectively).

## Results

### **Granulysin is expressed in V*δ*1^+^ and V*δ*2^+^** *γ* ***δ* T‐cell populations**

We first sought to confirm previous evidence that *γδ* T cells express granulysin when in a resting state.^20^ We assessed the intracellular expression of granulysin within this cell population and compared it to that observed within NK cells and CD8^+^ *αβ* T cells, previously shown to express granulysin constitutively and following an activation signal, respectively.^21^, ^22^ We then further separated the peripheral blood *γδ* T‐cell population into V*δ*1^+^ and V*δ*2^+^ subpopulations, and assessed the resting state expression of granulysin in each subpopulation.

**Figure 1:**
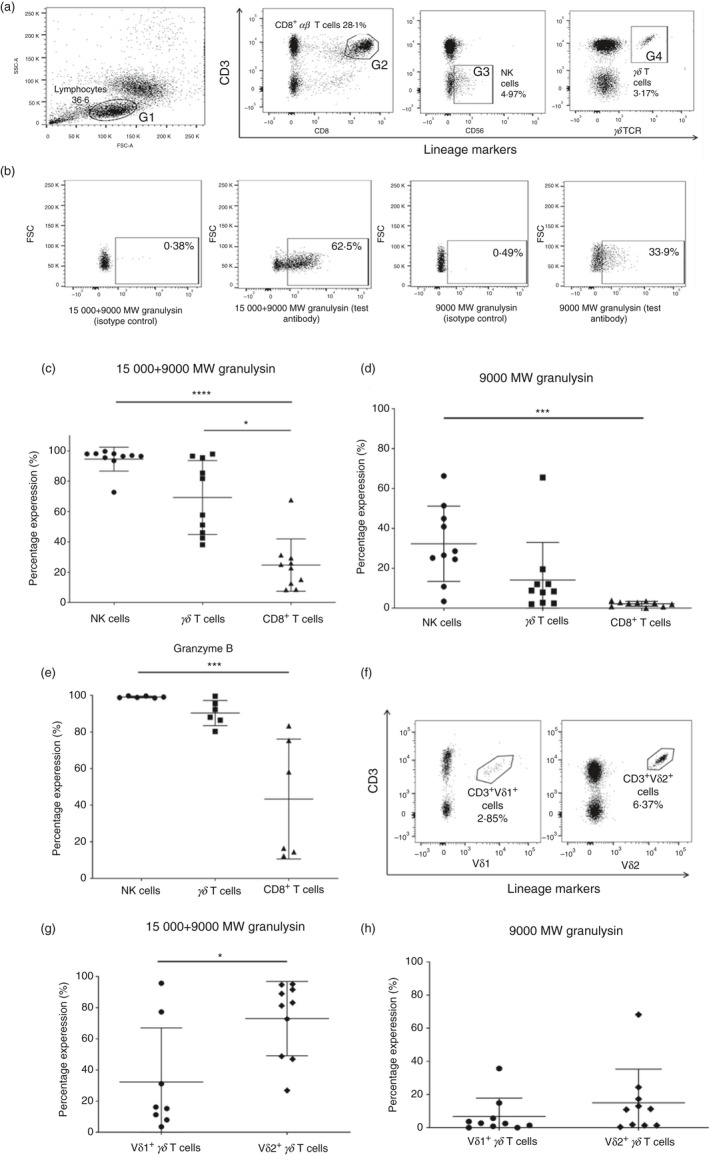
Resting *γδ* T cells express granulysin. (a) Representative flow cytometry plots depicting the gating strategy used to identify cell populations of interest from peripheral blood mononuclear cells (PBMC) preparations. Lymphocytes were gated (G1) according to size (forward scatter; FSC) and granularity (side scatter; SSC). Within gate G1, CD8^+^ *αβ* T cells, natural killer (NK) cells and *γδ* T cells were further gated on using established lineage markers for these cells (G2–G4). (b) Following identification of each immune cell population, the percentage of cells expressing 15 000+ 9000 MW granulysin or 9000 MW granulysin was determined by flow cytometry (expression within *γδ* T‐cell populations depicted). (c–e) Percentage expression of 15 000+ 9000 MW granulysin, 9000 MW granulysin, and granzyme B within populations of CD8^+^ *αβ* T cells, NK cells and *γδ* T cells, as determined by flow cytometry. (f) Gating strategy used to differentiate between V*δ*1^+^ *γδ* T cells and V*δ*2^+^ *γδ* T cells. (g,h) Percentage expression of 15 000+ 9000 MW and 9000 MW granulysin within populations of V*δ*1^+^ *γδ* T cells and V*δ*2^+^ *γδ* T cells as determined by flow cytometry. Data shown are obtained from between six and ten independent experiments using PBMC from ten individual donors, with error bars (SD). Differences between groups were assessed by one‐way analysis of variance. *P < 0·05. ***P < 0·001 ****P < 0·0001.

Flow cytometry was used to identify each immune cell population within PBMC preparations (Fig. 1a) and to determine the frequency of granulysin expression within these cells. Two antibodies were used to distinguish between total granulysin (hereafter referred to as 15 000+ 9000 MW granulysin) expression, and 9000 MW granulysin expression (Fig. 1b). Although the 9000 MW isoform of granulysin is produced through cleavage of the 15 000 MW precursor and therefore exhibits identical epitopes, the antibody used here to identify 9000 MW granulysin has been previously cited in the literature to have higher affinity for the cleaved 9000 MW granulysin isoform, compared with the full‐length 15 000 MW precursor ^19^, ^23^, ^24^

Natural killer cell populations had the highest percentage of cells expressing both isoforms of granulysin, and CD8^+^ *αβ* T cells had the lowest (Fig. 1c,d). Expression of granulysin, and granzyme B within *γδ* T‐cell populations, was most similar to that observed within NK cell populations (Fig. 1e). The percentage of V*δ*2^+^ *γδ* T cells found to be expressing either isoform of granulysin was analogous to that observed within *γδ* T‐cell populations as a whole, whereas very few V*δ*1^+^ *γδ* T cells were observed to express either isoform of granulysin (Fig. 1f–h).

Taken together, these results show that peripheral blood *γδ* T cells, and in particular those cells expressing a V*δ*2 chain, express both isoforms of granulysin when in a resting state, in a manner most comparable to NK cells of the innate immune system.

### *γδ* **T‐cell stimuli can increase the expression of intracellular 9000 MW granulysin**

We and others have shown that V*δ*2^+^ *γδ* T cells within PBMC preparations can be activated in response to short‐term treatment with ZA and BCG.^25^, ^26^ However, the ability of these stimuli to cause changes to the intracellular expression of granulysin within this cell population remains to be determined. We therefore conducted experiments to investigate whether 24 hr of stimulation with these reagents could cause an increase in the intracellular expression of granulysin in V*δ*2^+^ *γδ* T‐cell populations present within PBMC preparations.

**Figure 2:**
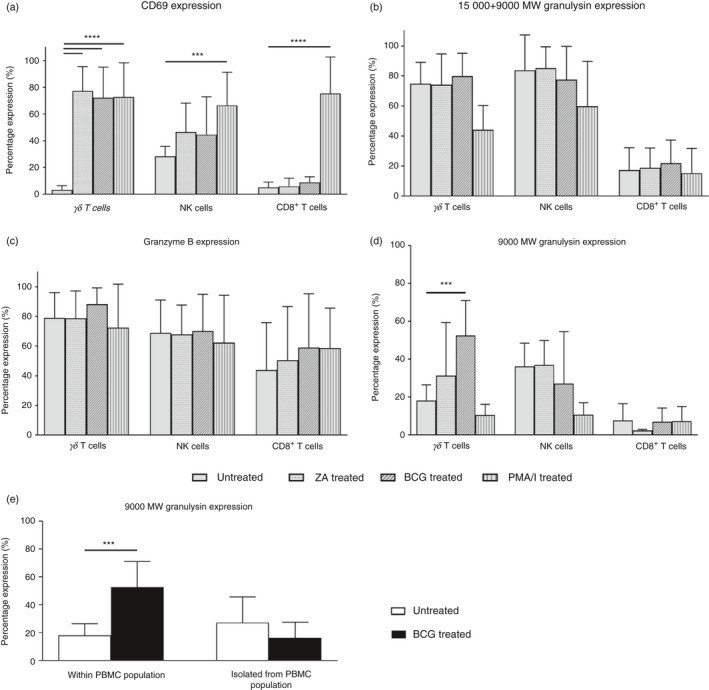
Stimulation of V*δ*2^+^ *γδ* T cells with bacillus Calmette–Guérin (BCG) causes an increase in the intracellular expression of 9000 MW granulysin. (a–d) The percentage of V*δ*2^+^ *γδ* T cells, natural killer (NK) cells and CD8^+^ T cells expressing early activation marker CD69, 15 000+ 9000 MW granulysin, granzyme B and 9000 MW granulysin following 24 hr of peripheral blood mononuclear cell (PBMC) stimulation with zoledronic acid (ZA), BCG or phorbol 12‐myristate 13‐acetate/ionomycin (PMA/I), as determined by flow cytometry. (e) The percentage of V*δ*2^+^ *γδ* T cells expressing 9000 MW granulysin following either 24 hr of PBMC stimulation with BCG, or following isolation of this cell population from unstimulated PBMC, and subsequent stimulation of these isolated V*δ*2^+^ *γδ* T cells for 24 hr. Data shown are mean values obtained from six independent experiments using PBMC from six individual donors, with error bars (SD). Statistics refer to the differences between treatment group and untreated group, and were assessed by one‐way analysis of variance. ***P < 0·001. ****P < 0.0001.

Twenty‐four hours of stimulation with ZA or BCG did not cause any marked expansion of V*δ*2^+^ *γδ* T cells within the PBMC population (not shown). However, an increase in the expression of activation marker CD69 on this cell population was observed, and was comparable to that seen following stimulation with PMA and ionomycin, known to cause activation of this cell type (Fig. 2a). Activation seen in response to either ZA or BCG was not observed within populations of CD8^+^ T cells or NK cells (Fig. 2a).

The percentage of V*δ*2^+^ *γδ* T cells expressing either 15 000+ 9000 MW granulysin or granzyme B was not changed in response to ZA or BCG stimulation (Fig. 2b,c). Interestingly, we observed a statistically significant increase in the percentage of V*δ*2^+^ *γδ* T cells expressing 9000 MW granulysin when PBMC were treated with BCG for 24 hr (Fig. 2d). A small increase in expression of this isoform was also observed following stimulation with ZA, although this was not statistically significant. This increase in 9000 MW granulysin expression on BCG treatment was not seen within populations of NK cells or CD8^+^ *αβ* T cells (Fig. 2d) or when isolated populations of V*δ*2^+^ *γδ* T cells were stimulated with BCG (Fig. 2e). This suggests that this response is specific to V*δ*2^+^ *γδ* T‐cell populations, and furthermore confirms previous literature suggesting that the involvement of additional immune cell populations is crucial to BCG‐induced stimulation of V*δ*2^+^ *γδ* T‐cell populations.^26^

Taken together, these data show that V*δ*2^+^ *γδ* T cells present within PBMC populations are activated by ZA or BCG, but only BCG stimulation causes a change in the expression of intracellular granulysin within this cell population.

### **Granulysin is released from V*δ*2^+^** *γ* ***δ* T cells in response to tumour**

**Figure 3:**
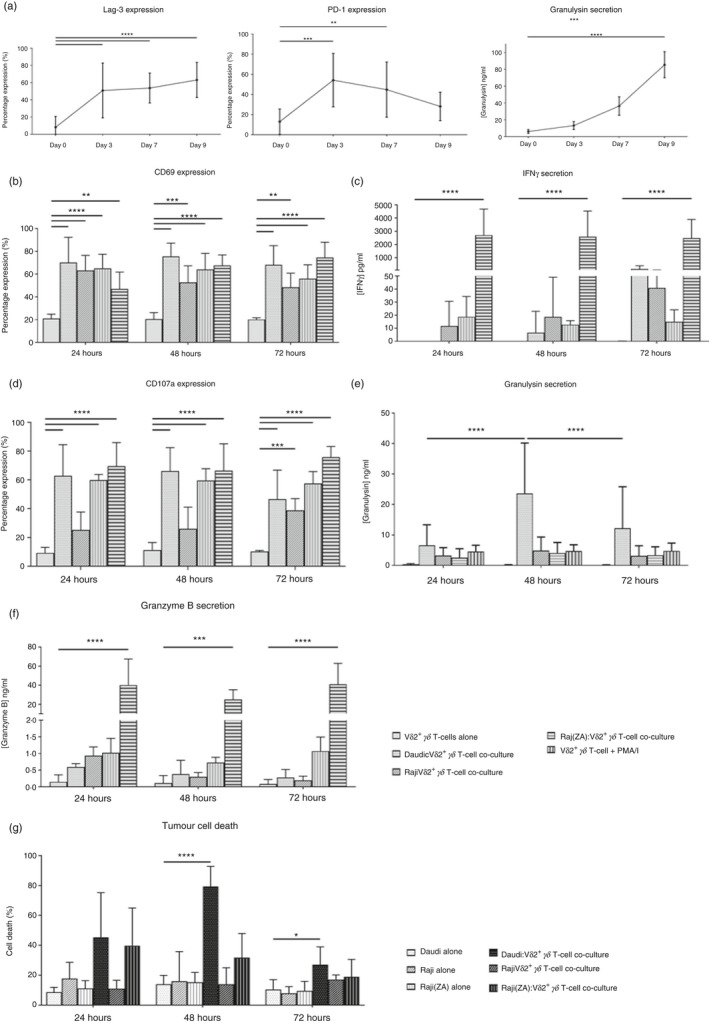
V*δ*2^+^ *γδ* T cells release granulysin in response to tumour. (a) The expression of exhaustion markers PD‐1 and Lag‐3 on, and the secretion of granulysin by, V*δ*2^+^ *γδ* T cells during the 9‐day expansion process. (b) The percentage of V*δ*2^+^ *γδ* T cells to express early activation marker CD69 following 24, 48 or 72 hr of culture with Daudi cells, Raji cells or Raji cells pre‐treated for 24 hr with 5 μm zoledronic acid (ZA), as determined by flow cytometry. (c) The concentration of interferon‐*γ* (IFN‐*γ*) found within supernatants taken from 24, 48 or 72 hr co‐culture of V*δ*2^+^ *γδ* T cells with tumour cell lines, as determined by ELISA. (d) The percentage of V*δ*2^+^ *γδ* T cells to express degranulation marker CD107a following 24, 48 or 72 hr of culture with Daudi cells, Raji cells or Raji cells pre‐treated for 24 hr with 5 μm ZA, as determined by flow cytometry. (e) The concentration of granulysin found within supernatants taken from 24, 48 or 72 hr co‐culture of V*δ*2^+^ *γδ* T cells with tumour cell lines, as determined by ELISA. (f) The concentration of granzyme B found within supernatants taken from 24, 48 or 72 hr co‐culture of V*δ*2^+^ *γδ* T cells with tumour cell lines, as determined by ELISA. (g) Percentage killing of tumour cells by V*δ*2^+^ *γδ* T cells following 24, 48 and 72 hr of culture, as determined by flow cytometry. Data shown are from six independent experiments, using V*δ*2^+^ *γδ* T cells from six individual donors, with error bars (SD). Differences between groups were assessed by two‐way analysis of variance comparing negative control (V*δ*2^+^ *γδ* T cells alone) with all other groups. *P < 0·05. **P < 0·01. ***P < 0·001. ****P < 0·0001.

We next designed experiments to investigate the release of granulysin from V*δ*2^+^ *γδ* T cells following culture with tumour cells. V*δ*2^+^ *γδ* T cells were isolated from PBMC pre‐treated for 9 days with IL‐2 and ZA to induce expansion of the V*δ*2^+^ *γδ* T‐cell population. Isolation of the V*δ*2^+^ *γδ* T‐cell population was achieved using MACS, and purity was determined by flow cytometry (see Supplementary material, Fig. S1). As expansion of V*δ*2^+^ *γδ* T cells requires prior activation of this cell type through treatment with ZA, cells were tested for markers of exhaustion throughout the expansion period to determine if their use would be feasible in subsequent co‐culture studies. We found an increase in the markers PD‐1 and Lag‐3 during the expansion process, but V*δ*2^+^ *γδ* T cells were still capable of secreting granulysin following the 9‐day expansion period, and so were determined to be viable for use in co‐culture studies (Fig. 3a).

V*δ*2^+^ *γδ* T cells were cultured with the B‐cell lymphoma lines Daudi and Raji, known to be sensitive and resistant to V*δ*2^+^ *γδ* T‐cell killing, respectively.^9^, ^27^ In addition, Raji cells were pre‐treated for 24 hr with ZA and subsequently washed before co‐culture, to render them more susceptible to V*δ*2^+^ *γδ* T‐cell killing.^28^ Preceding co‐culture, the ability of Daudi cells, Raji cells and Raji cells pre‐treated with ZA to produce the cytotoxic molecules investigated within this set of experiments was determined, and intracellular staining showed no expression of 15 000+ 9000 MW granulysin, 9000 MW granulysin, granzyme B or IFN‐*γ* within these cell populations (not shown). Following 24, 48 and 72 hr of culture, V*δ*2^+^ *γδ* T cells and co‐culture supernatants were harvested and used in flow cytometry and ELISA experiments, respectively.

Our data show that culture with Daudi cells, Raji cells and Raji cells pre‐treated with ZA caused a comparable increase in the expression of CD69 on V*δ*2^+^ *γδ* T cells when compared with that seen on V*δ*2^+^ *γδ* T cells cultured alone. This observation was seen regardless of the tumour cell line tested, suggesting that V*δ*2^+^ *γδ* T cells are activated by both Daudi and Raji tumour cell lines (Fig. 3b). Of note, we found that the percentage of V*δ*2^+^ *γδ* T cells expressing CD69 was approximately 60% lower than that observed when these cells were within a PBMC preparation (Fig. 2a). This is presumably because the isolated V*δ*2^+^ *γδ* T cells had been previously activated by ZA, during expansion of this cell population within PBMC preparations before isolation. Interestingly, although we observed activation of V*δ*2^+^ *γδ* T cells in response to all three tumour cell lines, we found only very small concentrations of IFN‐*γ* within supernatants taken from co‐culture of V*δ*2^+^ *γδ* T cells with all tumour cell lines tested (Fig. 3c).

Culture of V*δ*2^+^ *γδ* T cells with tumour cell lines caused an increase in the percentage of cells expressing CD107a (Fig. 3d). Although the percentages of V*δ*2^+^ *γδ* T cells expressing CD107a following culture with Daudi cells and Raji cells pre‐treated with ZA were comparable, CD107a expression on V*δ*2^+^ *γδ* T cells cultured with untreated Raji cells did not increase significantly above that observed in untreated cells, suggesting a lack of degranulation in response to this tumour cell type. Culture of V*δ*2^+^ *γδ* T cells with Daudi tumour cells caused the highest concentrations of granulysin released into co‐culture supernatants (Fig. 3e). However, contrary to our expectations, we found that the concentration of granzyme B within co‐culture supernatants did not follow this pattern. Instead, the concentrations of granzyme B found within co‐culture supernatants were notably lower than the concentrations of granulysin observed, and in fact, culture of V*δ*2^+^ *γδ* T cells with Daudi cells actually produced the lowest concentrations of granzyme B present within co‐culture supernatants (Fig. 3f). The peak in granulysin release from V*δ*2^+^ *γδ* T cells cultured with Daudi cells was observed following 48 hr of culture, and correlated with the time‐point at which the maximal killing of Daudi cells (79·3 ± 13·3%) was observed (Fig. 3g). Culture of V*δ*2^+^ *γδ* T cells with untreated Raji cells did not induce any secretion of granulysin above that produced by V*δ*2^+^ *γδ* T cells cultured alone, and also did not result in any marked increase in tumour cell death. Interestingly, although culture of V*δ*2^+^ *γδ* T cells with Raji cells pre‐treated with ZA did not appear to result in increased granulysin secretion in comparison to culture with untreated Raji cells, there was a substantial increase in killing of Raji cells pre‐treated with ZA following 24 hr of culture. This suggests that treating this cell line with ZA did result in some sensitization to V*δ*2^+^ *γδ* T‐cell killing.

Taken together, these data suggest that V*δ*2^+^ *γδ* T cells are activated by tumour cells, subsequently resulting in degranulation, granulysin release and cell death. However, this appears to occur without concomitant IFN‐*γ* and granzyme B release.

### Recombinant 15 000 MW granulysin can induce a mature phenotype in DC

**Figure 4:**
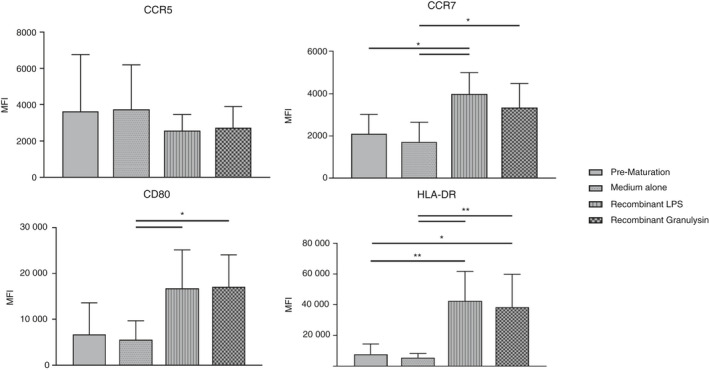
Granulysin can cause maturation of immature dendritic cells (DC). Changes in mean fluorescence intensity (MFI) of cell surface markers CD80, CCR7, CCR5 and HLA‐DR on monocyte‐derived immature DC following culture with 100 ng/ml lipopolysaccharide (LPS) or 66 nm recombinant 15 000 MW granulysin), as determined by flow cytometry. Treatment of cells with medium alone was included as a negative control. Data shown are the average taken from six individual donors. Differences between groups were assessed by two‐way analysis of variance comparing negative controls (pre‐maturation and medium alone) with all other groups. *P < 0·05. **P < 0·01.

Previous studies have shown the ability of recombinant 15 000 MW granulysin to cause maturation of immature DC,^17^, ^19^ and we sought here to replicate these findings. Immature DC were differentiated from isolated populations of peripheral blood CD14^+^ monocytes (see Supplementary material, Fig. S2), before being cultured for 24 hr in the presence of recombinant 15 000 MW granulysin. Culture of immature DC with LPS was used as a positive control of maturation, whereas medium alone was used as a negative control, and had no effect on maturation (Fig. 4). Flow cytometry and light microscopy were used to confirm the maturation of DC in response to each reagent tested.

Results showed that recombinant 15 000 MW granulysin was capable of maturation of immature DC (Fig. 4). Expression of CD80 and HLA‐DR, classical markers of maturation, increased significantly on cells following culture in the presence of recombinant 15 000 MW granulysin, and the mean fluorescence intensity observed was comparable to that seen following culture with the positive control of LPS. Additionally, expression of chemokine receptor CCR5, often expressed by immature, but not mature, DC, decreased following culture with both reagents. Conversely, expression of CCR7, the lymph node homing chemokine associated with maturation, increased. The expression of CD14 and CCR2 on DC before and after maturation was also determined, and did not alter (not shown).

Taken together, these findings suggest that recombinant 15 000 MW granulysin is capable of causing maturation of DC.

### Recombinant 15 000 MW granulysin can induce concentration‐dependent migration of immature and mature DC

As we had determined that 15 000 MW granulysin was capable of causing the maturation of immature DC, we next investigated if 15 000 MW granulysin could also cause migration of immature or mature DC. Previous literature has shown that 10 nm recombinant 15 000 MW granulysin can cause the migration of several immune cell populations, including DC.^16^, ^17^ We therefore sought to replicate DC migration in response to 10 nm recombinant 15 000 MW granulysin, and additionally tested migration in response to a higher concentration of 66 nm recombinant 15 000 MW granulysin. Ibidi μ‐migration assays and time‐lapse microscopy were used to follow the migration patterns of cells in response to each stimulus for 24 hr. Figure S3 (see Supplementary material) provides detailed methodology for determination of percentage migration in response to a stimulus. Recombinant RANTES was used as a positive control of migration for immature DC, and recombinant CCL19 was used as a positive control of migration for mature DC. Concentrations used for positive controls were based on manufacturer’s recommendation.

**Figure 5:**
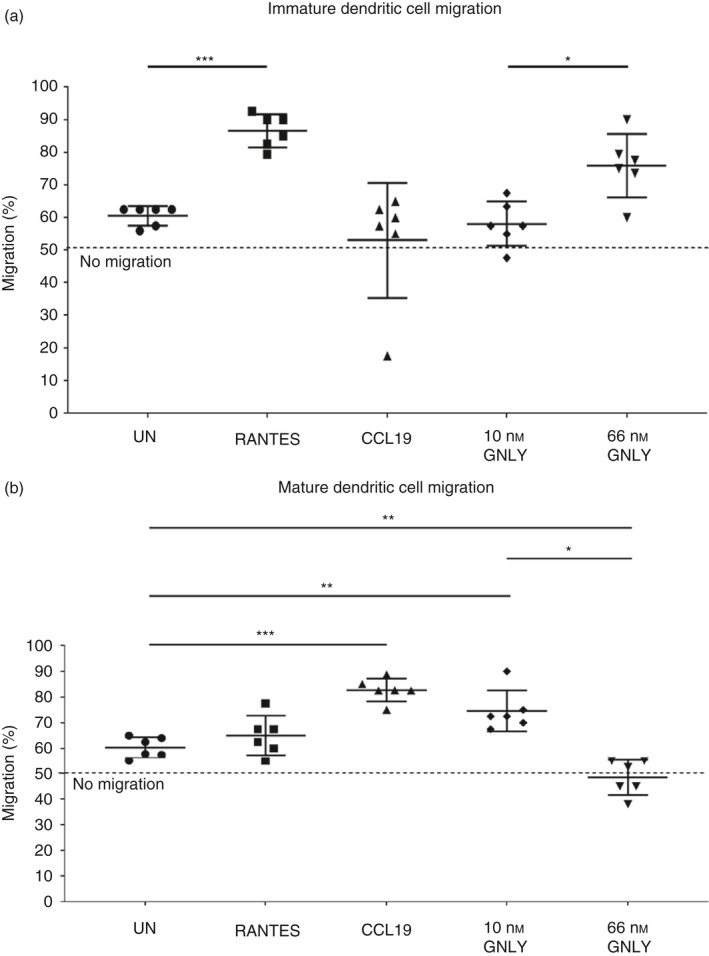
Recombinant 15 000 MW granulysin causes differential migration of immature and mature dendritic cells (DC). (a) The migration of immature DC in response to 10 or 66 nm recombinant 15 000 MW granulysin, as determined by Ibidi μ‐migration assays. 500 ng/ml recombinant RANTES was used as a positive control of immature DC migration, while 2 ng/ml recombinant CCL19 was used as a negative control. (b) The migration of mature DC in response to 10 or 66 nm recombinant 15 000 MW granulysin, as determined by Ibidi μ‐migration assays. 500 ng/ml recombinant RANTES was used as a negative control of mature DC migration, whereas 2 ng/ml recombinant CCL19 was used as a positive control. Concentrations used for positive and negative controls were based on manufacturer’s recommendation. Data shown are from six independent experiments using immature and lipopolysaccharide‐matured DC differentiated from the monocytes of six individual donors, with error bars (SD). Differences between groups were assessed using one‐way analyses of variance. *P < 0·05. **P < 0·01. ***P < 0·001. UN = untreated. GNLY = 15 000 MW granulysin.

In keeping with results obtained by Deng *et al*.,^16^ we found that although immature DC did not migrate in response to 10 nm recombinant 15 000 MW granulysin, this concentration of granulysin caused marked migration of mature DC (Fig. 5a,b). Interestingly, when the concentration of recombinant 15 000 MW granulysin was increased to 66 nm, mature DC no longer migrated towards this reagent, and in fact the percentage migration of these cells was determined to be less than that seen in response to the negative control of medium alone, indicating a movement away from this concentration of granulysin (Fig. 5c). In contrast, immature DC were found to migrate towards 66 nm recombinant 15 000 MW granulysin (Fig. 5a), and percentage migration was found to be comparable to the positive control of recombinant RANTES.

Taken together, these data suggest that recombinant 15 000 MW granulysin can cause the migration of immature and mature DC. In addition, results suggest that granulysin may differentially cause both migration and repulsion of matured DC, dependent on the concentration of granulysin.

## Discussion

The remit of this study was to determine whether V*δ*2^+^ *γδ* T cells released granulysin in response to a tumour target, and to investigate the functional consequences of this response. In this paper, we present evidence that V*δ*2^+^ *γδ* T cells express granulysin intracellularly in a constitutive manner, and release this molecule on culture with the tumour cell lines Daudi and Raji, albeit to differing degrees. In addition, we show that 15 000 MW granulysin can cause the maturation of immature DC, and further propose that 15 000 MW granulysin may have a dual capacity to cause both the chemotaxis of immature DC and the fugetaxis of mature DC, in a concentration‐dependent manner.

Often referred to as ‘the bridge between the innate and adaptive immune systems’, it has been established that V*δ*2^+^ *γδ* T cells possess properties of both innate and adaptive immune cells. Despite being a relatively small immune cell subset, V*δ*2^+^ *γδ* T cells have been shown to respond rapidly to tumour, due to their ability to recognize targets without prior antigen processing and presentation. In this way, V*δ*2^+^ *γδ* T cells make a crucial contribution to the immune response to cancer. In fact, research by Gentles *et al*.^29^ into the association of infiltrating immune cell subsets with prognostic outcomes showed that *γδ* T cells were ranked as the highest indicator of a favourable outcome for 25 different malignancies and 14 solid tumours. Additionally, several studies have cited the involvement of V*δ*2^+^ *γδ* T cells activated with BCG in the regression of tumour. For example, Takeuchi and colleagues determined that production of IL‐17 by *γδ* T cells following BCG inoculation in bladder cancer was responsible for the subsequent recruitment of neutrophils required for an anti‐tumour response.^30^ More recently, substantial V*δ*2^+^ *γδ* T‐cell infiltration has been identified in metastatic melanoma lesions following intralesional injection of BCG. Yang *et al*.^31^ observed an increase in CXCL9, CXCL10 and CXCL11, in addition to increased expression of butyrophilin 3A1 in these lesions following treatment, which they hypothesized resulted in the attraction and subsequent activation of intralesional V*δ*2^+^ *γδ* T cells. Interestingly, injection of these lesions with BCG led to a 50% regression in tumour size, which may be linked to the increased number of responding V*δ*2^+^ *γδ* T cells.^31^ This emerging correlation between positive cancer outcomes and the presence of activated V*δ*2^+^ *γδ* T cells led us to further investigate the ways in which this cell population could be activated to release cytotoxic molecules, and the functional effects of this with regard to tumour cell killing.

In this study, we show that *γδ* T cells do not require a stimulatory signal to express 15 000 MW granulysin intracellularly, and only express 9000 MW granulysin to a high level, at least in our hands, following BCG stimulation. These findings show similarities of *γδ* T cells with both NK cells of the innate immune system, and CD8^+^ *αβ* T cells of the adaptive immune system. NK cells have been shown to express both isoforms of granulysin constitutively, and CD8^+^ *αβ* T cells have been shown to express granulysin only 3–4 days after activation.^22^, ^32^ We also found that the percentage of V*δ*2^+^ *γδ* T cells expressing each isoform of granulysin was comparable to that observed within the *γδ* T‐cell population as a whole. The presence of intracellular granulysin within this subpopulation before recognition of tumour and subsequent activation may allow a more rapid release of granulysin into the surrounding environment when activation of the cell does occur.

Our findings within this study have confirmed previous evidence that V*δ*2^+^ *γδ* T cells can be activated by both ZA and BCG, as long as additional immune cell populations are present.^25^, ^26^ However, only activation of PBMC with BCG was able to cause a change in the percentage of V*δ*2^+^ *γδ* T cells expressing granulysin. It is not surprising that stimulation of V*δ*2^+^ *γδ* T cells caused an increase in the expression of 9000 MW granulysin only; this is the cytotoxic isoform of granulysin, which has been shown to increase within other T‐cell populations following activation.^32^ Interestingly, previous studies have shown an increase in the expression of granulysin within populations of CD8^+^ *αβ* T cells and CD4^+^ *αβ* T cells following BCG vaccination of neonates, so it is perhaps not unexpected that this effect is also seen in populations of V*δ*2^+^ *γδ* T cells.^33^ However, a significant increase in intracellular 9000 MW granulysin expression was not observed following stimulation of V*δ*2^+^ *γδ* T cells with ZA. This could be a result of differences in the efficacy of IPP produced in response to ZA, and HMBPP produced by BCG infection.^8^ Alternatively, the stimulation of PBMC with BCG has been shown to cause activation of other populations of immune cells.^34^, ^35^ This activation may deliver a co‐stimulatory signal to V*δ*2^+^ *γδ* T cells, which is necessary for the up‐regulation of 9000 MW granulysin within these cells, and which is not delivered following stimulation with ZA, and highlights the requirement for other immune cell populations in the activation of V*δ*2^+^ *γδ* T cells in response to these reagents.

For this reason, we sought to confirm that isolated V*δ*2^+^ *γδ* T cells could release granulysin in response to tumour without additional activation. We showed that *in vitro*, V*δ*2^+^ *γδ* T cells released substantial amounts of granulysin in response to culture with Daudi tumour cells, known to be sensitive to V*δ*2^+^ *γδ* T‐cell killing, and that they could also release this molecule, albeit to a lesser degree, in response to Raji cells (resistant to V*δ*2^+^ *γδ* T‐cell killing) and Raji cells pre‐treated with ZA. It is interesting to note that although co‐culture caused an increase in the production of the cytotoxic molecule granulysin by V*δ*2^+^ *γδ* T cells, production of the classical cytotoxic cytokine IFN‐*γ* remained low over all conditions. This is in contrast to previous evidence, which shows that V*δ*2^+^ *γδ* T cells express IFN‐*γ* following activation, and that V*δ*2^+^ *γδ* T cells deficient in IFN‐*γ* are less likely to be able to kill tumour.^36^, ^37^ A potential explanation for this finding is that both Daudi and Raji cells express receptors for IFN‐*γ*, suggesting that any IFN‐*γ* released by V*δ*2^+^ *γδ* T cells in response to tumour may be taken up by the tumour cells themselves, and as such will not be present within supernatants to be detected by ELISA.^38^, ^39^ The fact that the highest concentrations of IFN‐*γ* detected by ELISA were found within supernatants taken from the co‐cultures of V*δ*2^+^ *γδ* T cells with Daudi cells suggests an excess of IFN‐*γ* produced by V*δ*2^+^ *γδ* T cells that cannot be taken up by the Daudi cells.

It is interesting that the pre‐treatment of Raji cells with ZA did not increase the release of granulysin to a level comparable to that seen following culture of V*δ*2^+^ *γδ* T cells with Daudi cells, despite increasing the amount of tumour cell death. The addition of ZA to Raji cells has been previously shown to cause an increase in IPP expression, and so an increase in V*δ*2^+^ *γδ* T‐cell cytotoxicity. It is possible that too low a concentration of ZA was used in the experiments detailed here. Although we used 5 µm ZA to induce sensitization of Raji cells to V*δ*2^+^ *γδ* T‐cell killing, previous evidence by Idrees *et al*. has shown that inhibition of farnesyl pyrophosphate synthase was not observed within Raji cells until a concentration of 1 mm ZA was added to cells *in vitro*. In addition, cytotoxicity of V*δ*2^+^ *γδ* T cells, as characterized by production of tumour necrosis factor‐*α*, was also not observed below this concentration of ZA.^40^ Despite this, our data suggest that granulysin may be released by V*δ*2^+^ *γδ* T cells that infiltrate and recognize tumours *in vivo*, which are sensitive to killing by this cell type.

We next investigated the role of granulysin in the maturation of DC, and showed that recombinant 15 000 MW granulysin was capable of causing the maturation of immature DC in a manner similar to that seen in response to recombinant LPS. Granulysin has been described as an immune alarmin,^17^ and several other alarmins have been shown to cause the maturation of immature DC. For example, research by Dumitriu *et al*.^41^ showed that high mobility group box 1 caused maturation of DC, characterized by an increase in expression of CCR7. We found that high concentrations of recombinant 15 000 MW granulysin were also able to cause migration of immature DC. This is of interest as it suggests that in a physiological setting, granulysin released by V*δ*2^+^ *γδ* T cells in response to a tumour target may contribute to the influx of immature DC to the tumour site. However, perhaps more noteworthy is the observation that recombinant 15 000 MW granulysin appears to cause both the chemotaxis and fugetaxis of matured DC dependent on concentration. Low concentrations of granulysin were found to cause a marked migration of mature DC, whereas high concentrations of granulysin induced a movement of these cells away from this molecule. Research has shown evidence of this phenomenon previously. Tharp determined that whether a neutrophil migrated towards or away from IL‐8 was dependent on the absolute concentration of this molecule.^42^ Using microfluidic linear gradient generators and time‐lapse microscopy, results showed that at concentrations of 120 nm, neutrophils were seen to migrate towards IL‐8, whereas at concentrations of 1·2 μm, neutrophils displayed potent fugetaxis.^42^ A similar phenomenon has been shown for the ability of stromal cell derived factor (SDF) ‐1 to cause the attraction and repulsion of T cells. At 100 ng/ml, SDF‐1 caused chemoattraction of both naive and memory CD4^+^ and CD8^+^ *αβ* T cells, whereas higher concentrations of 10 μg/ml SDF‐1 caused repulsion of these cells.^43^

To further assess the contribution of granulysin released by V*δ*2^+^ *γδ* T cells in response to tumour in the maturation and migration of DC, it would be important to next determine whether we could replicate our findings using supernatants taken from the co‐culture of these cells with tumour targets. We have conducted preliminary experiments and observed that supernatants taken from the co‐culture of V*δ*2^+^ *γδ* T cells and Daudi cells could effectively induce both maturation of immature DC, and the migration of these cells towards the supernatant source (see Supplementary material, Figs S4 and S5A). Interestingly, we were also able to replicate the differential migration of mature DC towards or away from granulysin, depending on the concentration of this molecule present in the supernatants tested. As can be seen from Fig. S5B (see Supplementary material), mature DC migrated towards supernatants taken from the co‐culture of V*δ*2^+^ *γδ* T cells with Raji cells, which contained low concentrations of granulysin (an average of 4·85 ng/ml). However, these cells migrated away from supernatants taken from the co‐culture of V*δ*2^+^ *γδ* T cells with Daudi cells, containing higher concentrations of this molecule (an average of 28·60 ng/ml). The inclusion of a granulysin blocking antibody, before the addition of the supernatants to cultures of DC, would allow definitive confirmation of the involvement of 15 000 MW granulysin in the effects observed, and this would form the basis of future work.

Our data suggest that V*δ*2^+^ *γδ* T cells, through the production and release of granulysin, may be involved in orchestrating adaptive immunity against tumour. Through the release of granulysin, this cell population may contribute to the arrival of immature DC populations to a site of tumour, and may then further contribute to the migration of matured DC away from the tumour site, and towards lymph nodes to activate the adaptive immune response. We believe this to be an interesting facet of the anti‐tumour response of V*δ*2^+^ *γδ* T cells, and therefore worthy of further investigation.

## Disclosure

The authors declare no financial or commercial conflicts of interest.

## Author contributions

ELS performed the experiments. MDB‐S and AGD designed the study, and DW Fowler and J Caron contributed to the design of experiments. All authors contributed ideas to the study and experimental design. ELS wrote the manuscript, and all authors proofread the manuscript.

## Ethical approval

The use of human blood donors was approved by St George’s, University of London ethics committee (number SGREC16.0009) in October 2013.

## Supporting information


**Figure S1.** Representative gating strategy used to determine purity of isolated V*δ*2^+^ *γδ* T cells.
**Figure S2.** Representative gating strategy used to establish purity of CD14^+^ monocytes following isolation.
**Figure S3.** Example μ‐migration assay analysis.
**Figure S4.** Granulysin‐containing supernatants can cause maturation of immature dendritic cells.
**Figure S5.** Granulysin‐containing supernatants cause differential migration of immature and mature dendritic cells.

## Data Availability

The data that support the findings of this study are available from the corresponding author upon reasonable request.
