# Supplementary material for: The cytotoxic molecule granulysin is capable of inducing either chemotaxis or fugetaxis in dendritic cells depending on maturation: a role for Vδ2+ γδ T cells in the modulation of immune response to tumour?
Source: Immunology. 2020 Sep 16;161(3):245–58. doi: 10.1111/imm.13248 (PMC7576882; doi:10.1111/imm.13248)
Supplement: Supplementary file 1 — Figure S1. Representative gating strategy used to determine purity of isolated Vδ2+ γδ T cells. Figure S2. Representative gating strategy used to establish purity of CD14+ monocytes following isolation. Figure S3. Example μ‐migration assay analysis. Figure S4. Granulysin‐containing supernatants can cause maturation of immature dendritic cells. Figure S5. Granulysin‐containing supernatants cause differential migration of immature and mature dendritic cells. [file IMM-161-245-s001.docx]

# Supplemental figures


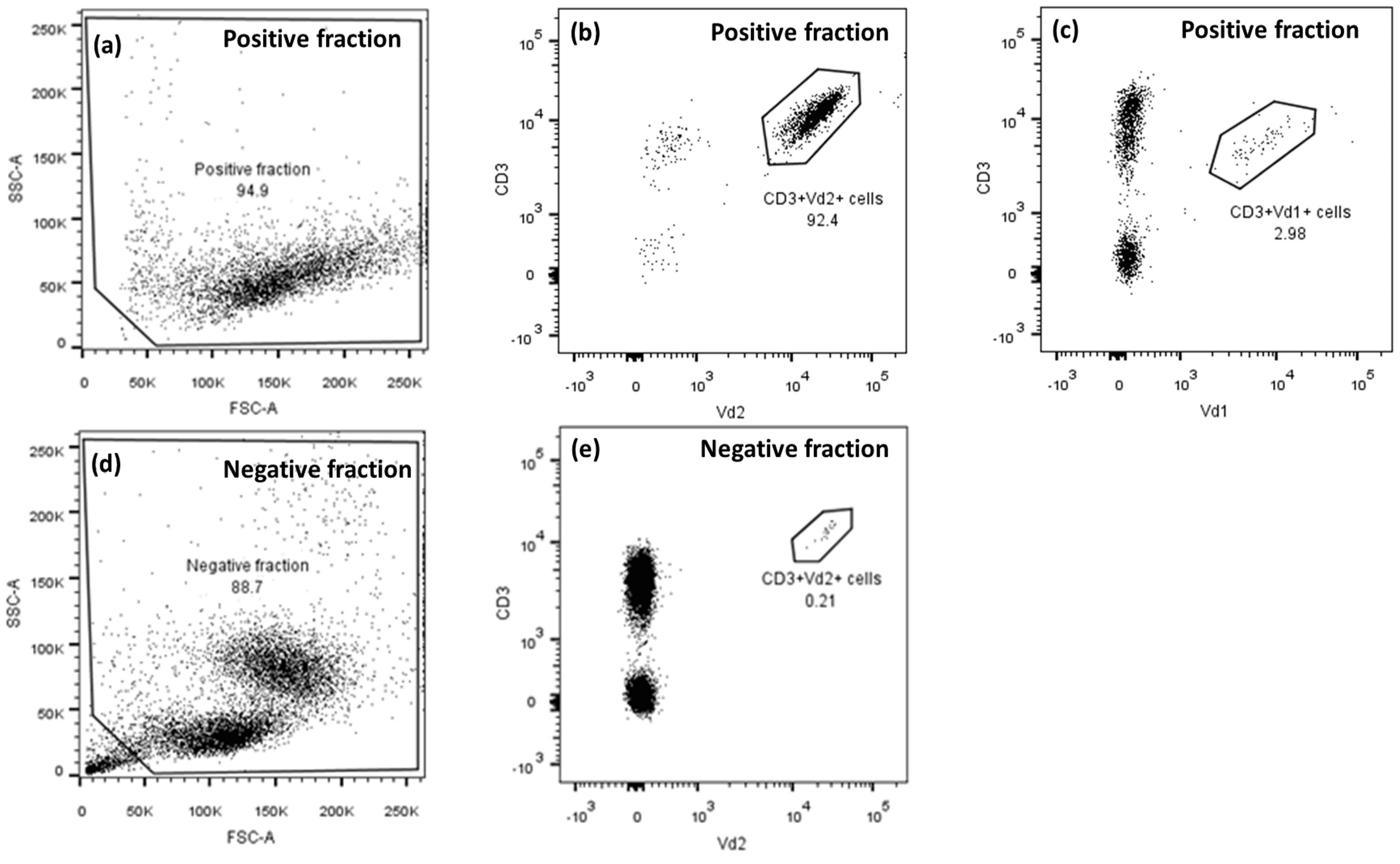


***Figure S1: Representative gating strategy used to determine purity of isolated Vδ2^+^ γδ T cells.***

*PBMC were isolated from whole blood samples taken from healthy volunteers, and treated with 5µM ZA and 15ng/ml (315U/ml) IL-2 for 9 days in order to expand populations of Vδ2^+^ γδ T cells. Following expansion culture, γδ T cells were isolated using MACS and negative selection, and the purity of γδ T cell populations following isolation determined by flow cytometry. The percentage of Vδ2^+^ γδ T cells within positive (a-c) and negative fractions (d-e) was established. Cells were plotted based on FSC and SSC, and a gate placed around all cells ((a) and (d)). Fluorochrome-conjugated antibodies specific for cell surface markers CD3 and Vδ2 were used to identify populations of Vδ2^+^ γδ T cells within each fraction ((b) and (e)). Within the positive fraction, the percentage of Vδ1^+^ γδ T cells was also identified using a fluorochrome-conjugated antibody specific for Vδ1, as the isolation process is not specific for Vδ2^+^ γδ T cells (c). If the combined percentage of Vδ1^+^ and Vδ2+ γδ T cells was over 90%, cells were used in subsequent co-culture experiments.*


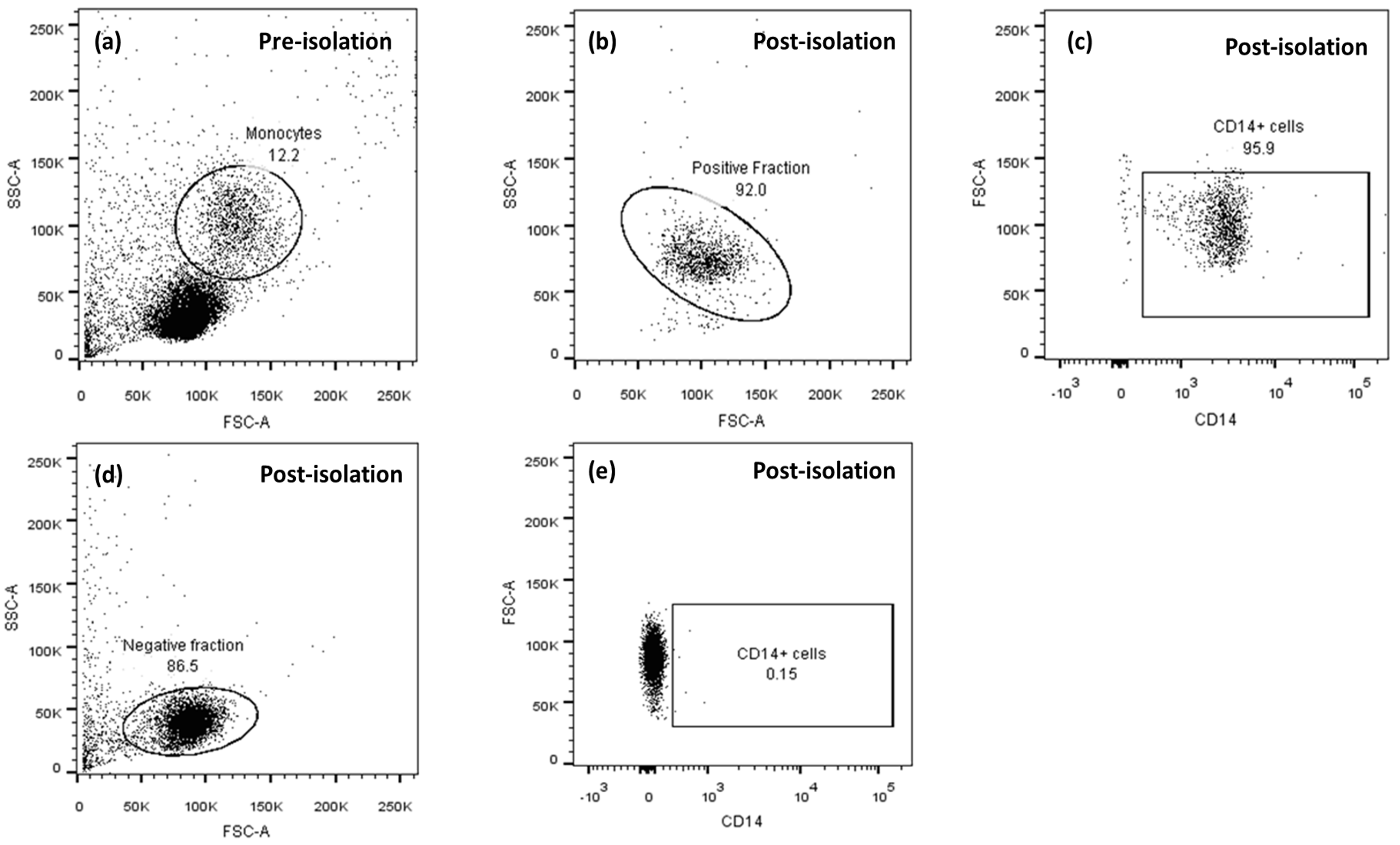


***Figure S2: Representative gating strategy used to establish purity of CD14^+^ monocytes following isolation****.*

*Populations of CD14^+^ monocytes were identified within PBMC preparations prior to isolation (a). Following isolation via positive selection and through MACS, cells present in the positive fraction were gated on (b), and the percentage of CD14^+^ cells within this fraction determined (c). In addition, the negative fraction (d) was also subjected to flow cytometry, and the percentage of CD14^+^ cells present within this fraction determined (e), in order to establish the efficiency of the isolation procedure.*

**S3: Example μ-migration assay analysis.**

Migration of immature and mature DC in response to granulysin was measured using Ibidi μ-migration assays. Photographs of cells present within each chemoattractant chamber were taken every 15 minutes during a 24 hour time period, using a time-lapse microscope. Following the end of the assay, images of the cells prior to commencement of the assay were used to choose 40 cells for tracking (S3.1).


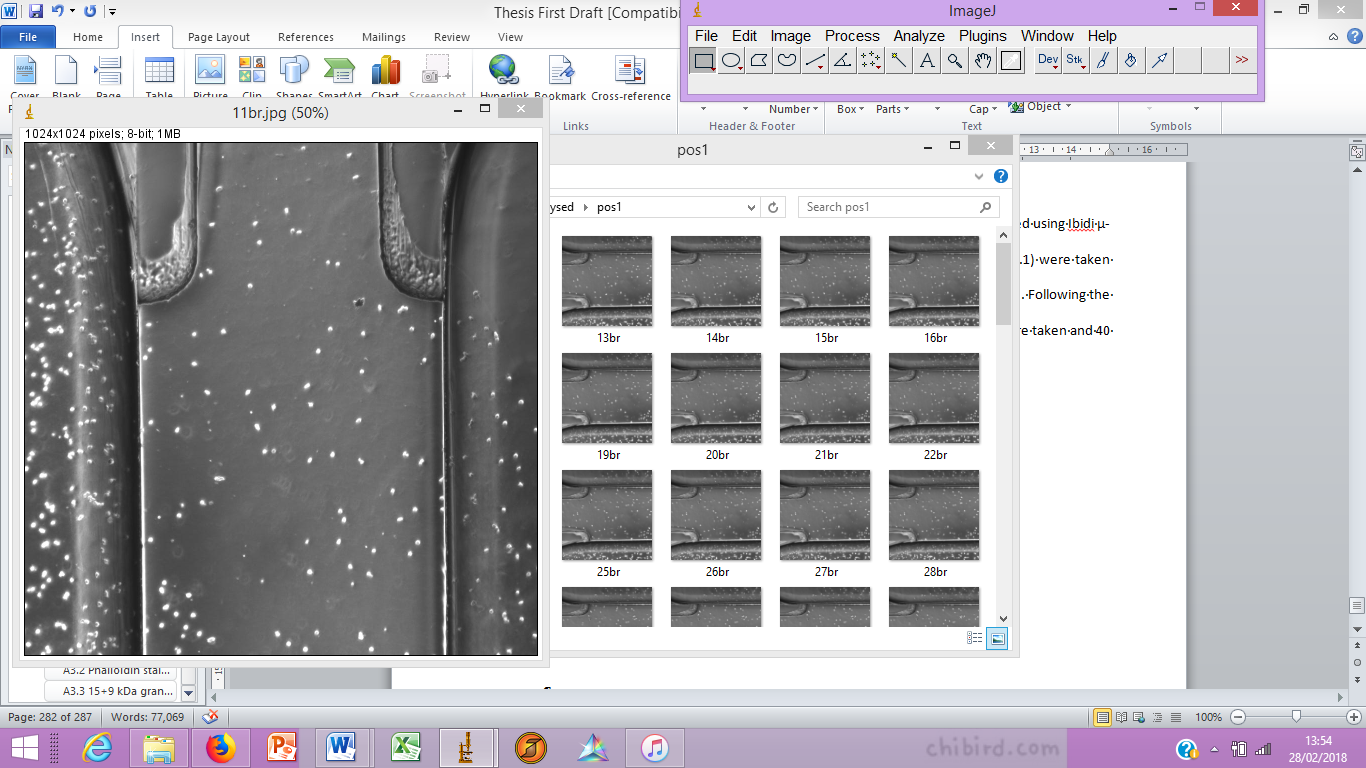


**CHEMOATTRACTANT**

**MEDIUM**

**12**

**13**

**20**

**14**

**18**

**17**

**19**

**10**

**8**

**5**

**4**

**6**

**3**

**7**

**2**

**1**

**16**

**11**

**15**

**9**


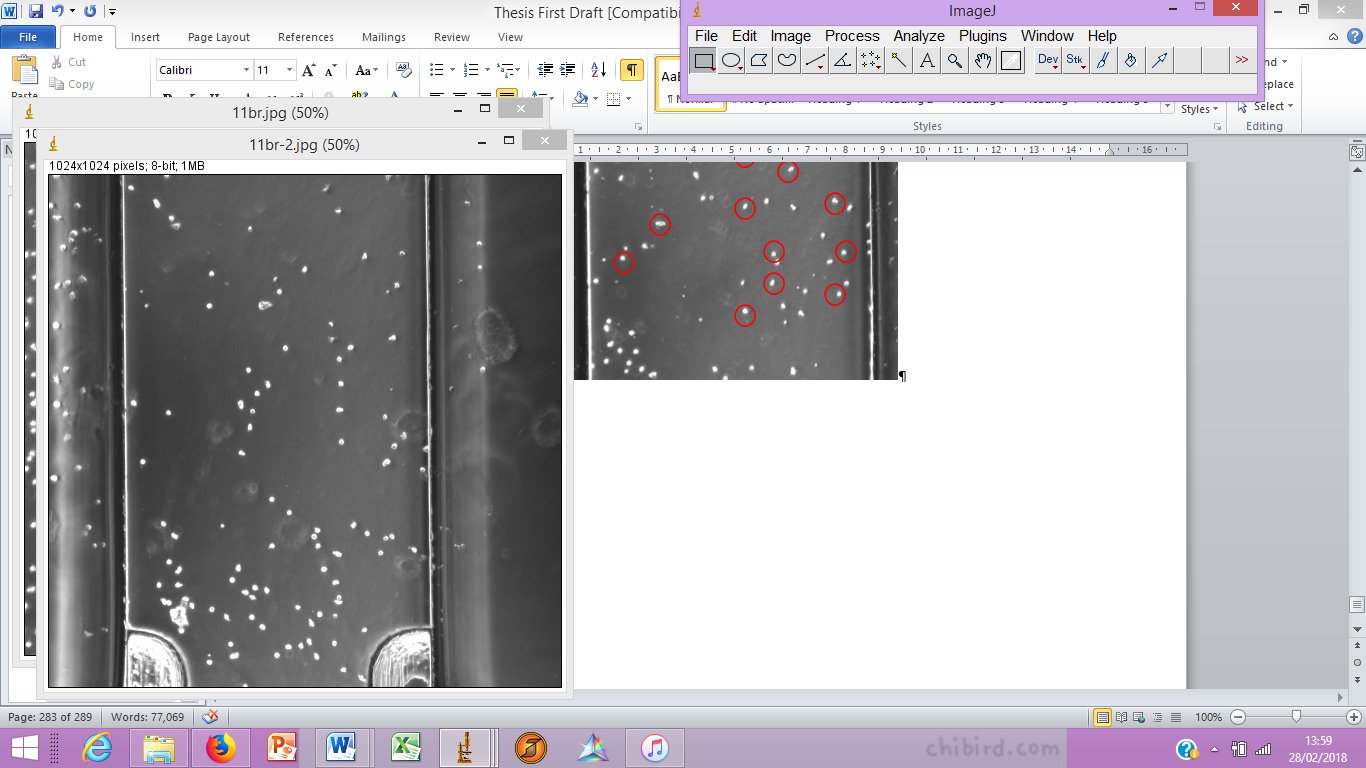


**S3.1: Representative photograph taken of one cell chamber of a μ-migration slide prior to commencement of the assay, and the 40 cells chosen for tracking.**

**33**

**21**

**37**

**23**

**29**

**28**

**25**

**30**

**34**

**31**

**35**

**36**

**40**

**26**

**38**

**39**

**32**

**27**

**24**

**22**

Once cells had been chosen, ImageJ software was used to manually track the migratory path of each selected cell. This was conducted through importing all images taken of each part of a chamber within a 24 hour run, and manually clicking through each image following the path of the selected cell. A plugin for ImageJ called manual tracking (S3.2) recorded the position of the cell on each image as images were clicked through. This was repeated for each of the 40 selected cells. A proportion of experiments were additionally tracked by an independent researcher, in order to ensure the validity of results obtained.


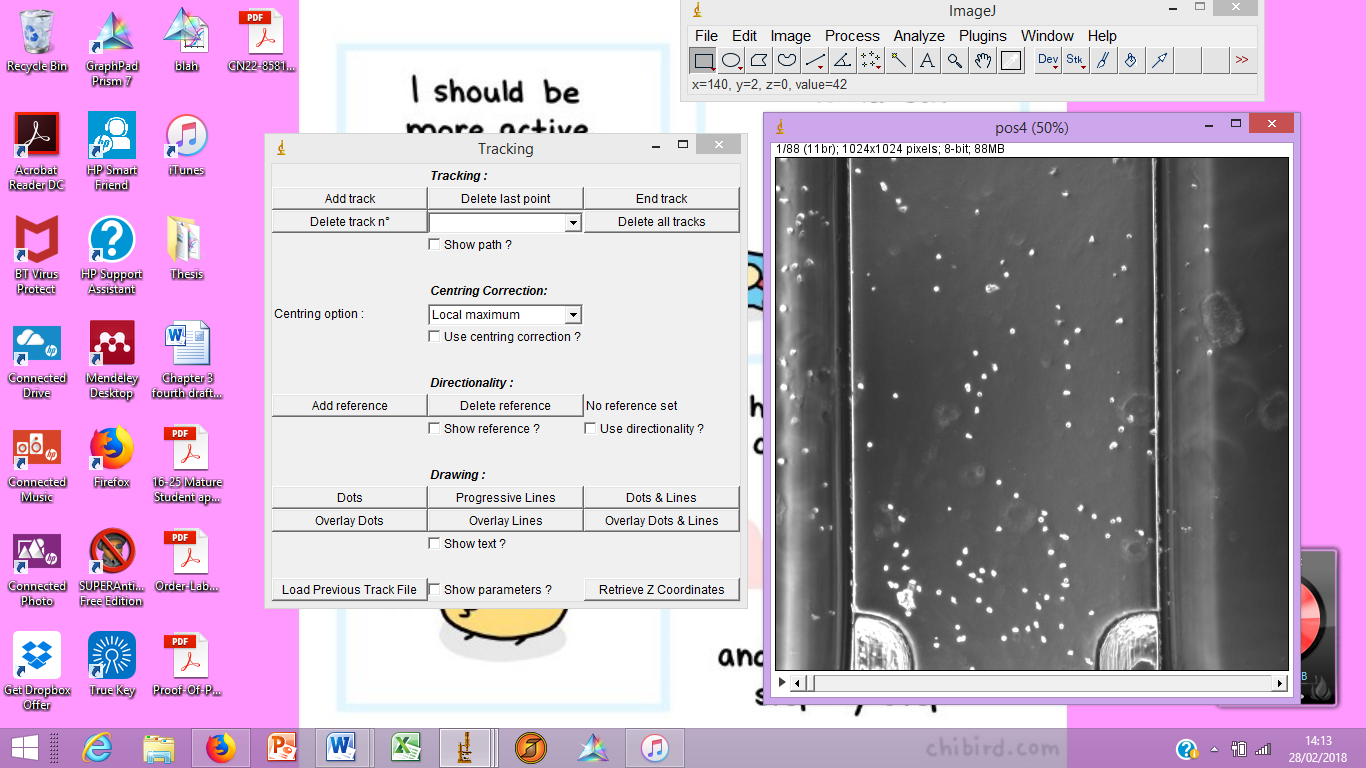


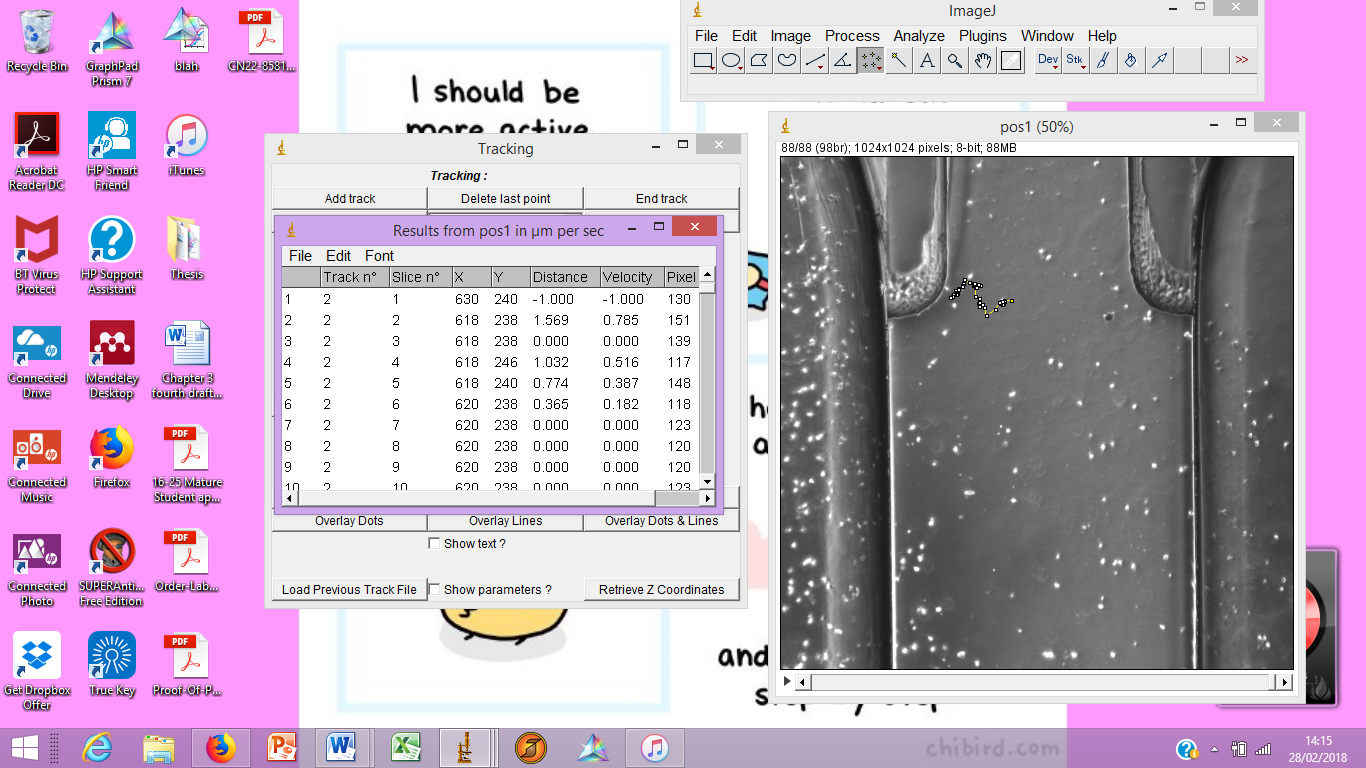


**S3.2: ‘Manual tracking’ plugin within ImageJ software used for tracking migratory path of cells.**


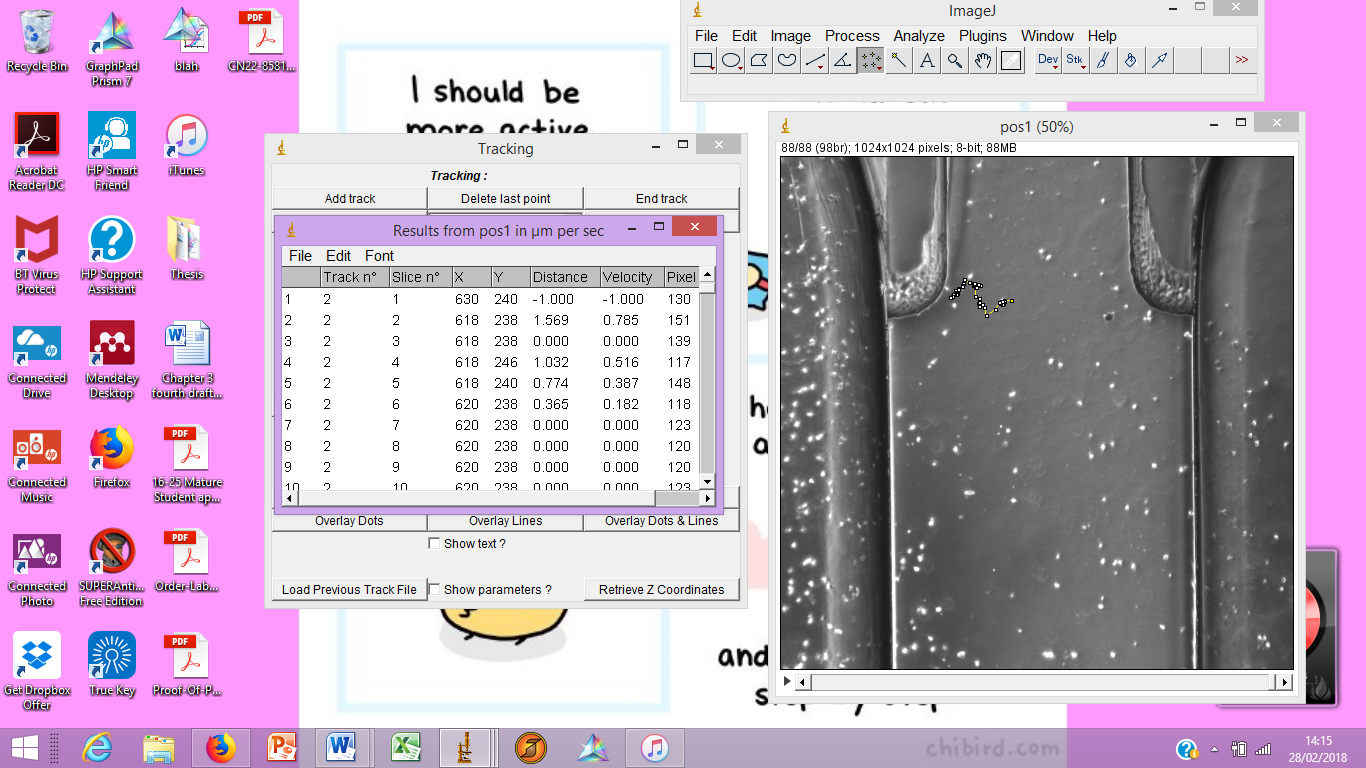


Direction of travel

**MEDIUM**

**CHEMOATTRACTANT**

**S3.3: Representative image showing a single migratory path of a cell manually tracked using the ‘manual tracking’ plugin within ImageJ software.**

Once all 40 cells had been tracked, a second plugin called ‘chemotaxis tool’ was utilised to determine the number of cells which had migrated towards the chemoattractant (S3.4).


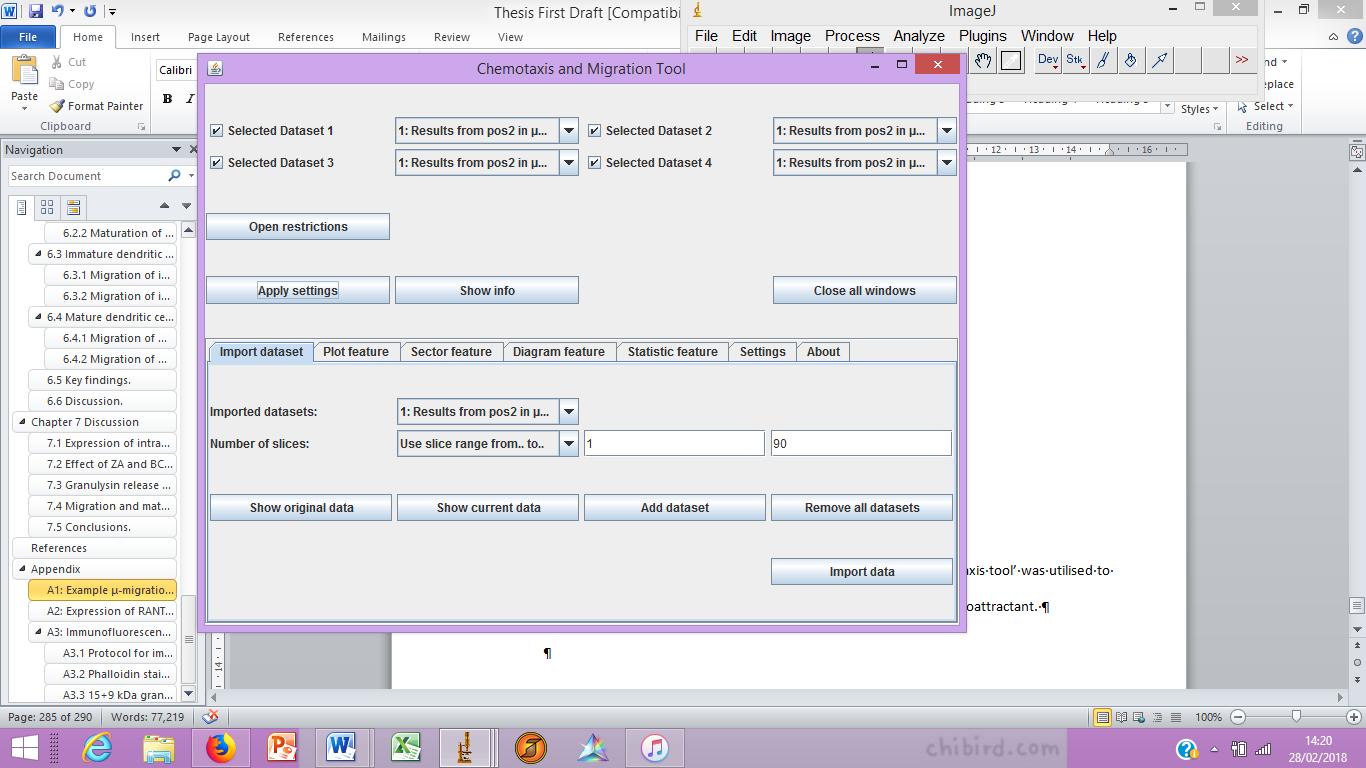


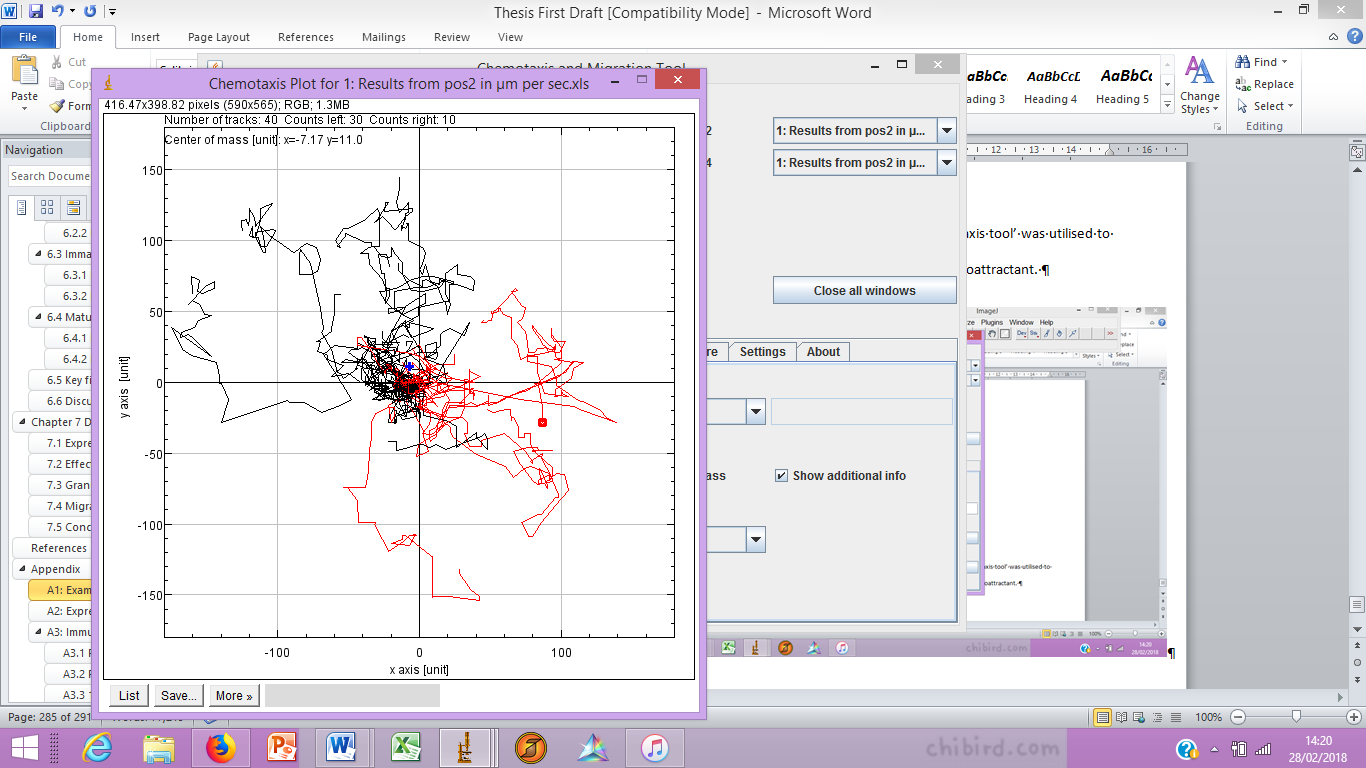


**S3.4: Representative graph plotted using the ‘chemotaxis tool’ plugin within ImageJ software, showing the migratory tracks of 40 cells, and the number of cells which had moved towards or away from the chemoattractant.**

In this case, 30 cells out of a total of 40 were determined to have moved towards the chemoattractant (black lines), while 10 cells had moved away from it (red lines). Therefore, a percentage migration of 75% was achieved in response to this chemoattractant.


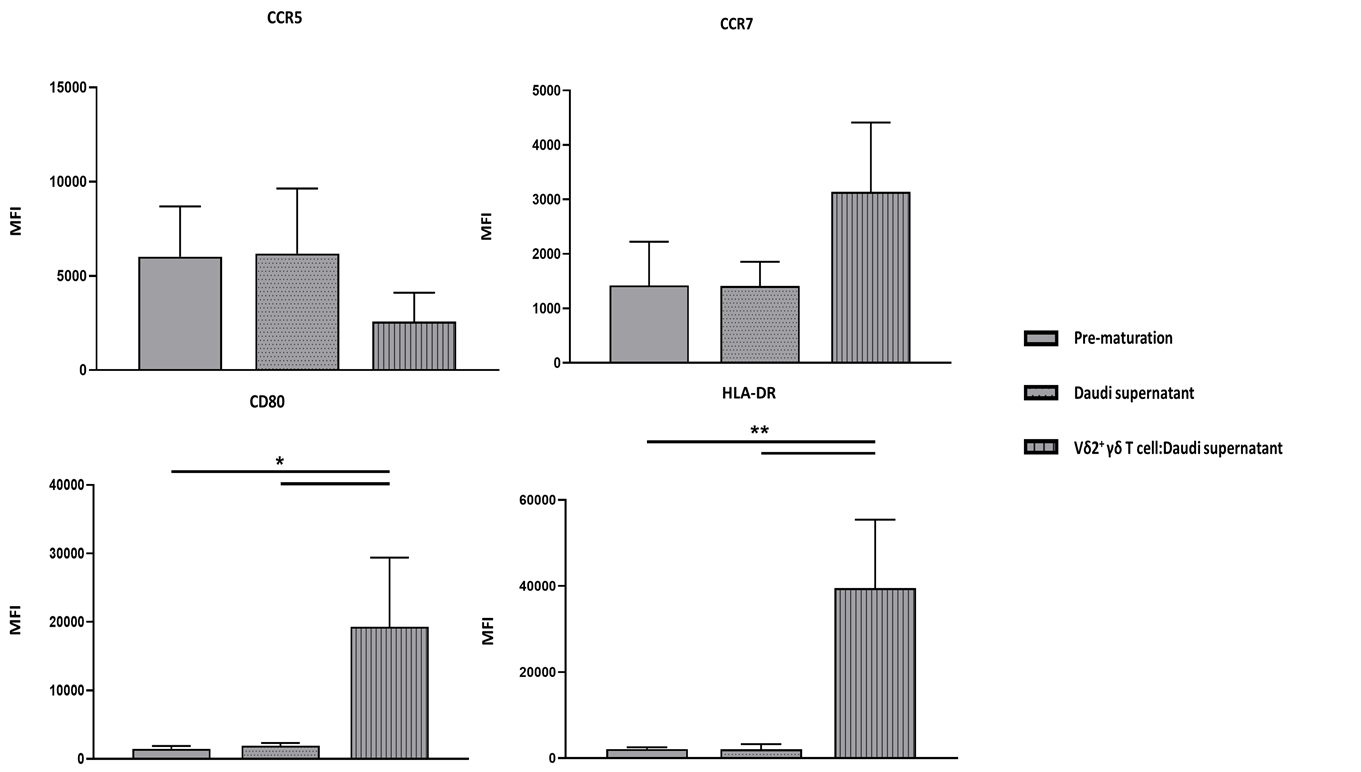


***Figure S4: Granulysin-containing supernatants can cause maturation of immature DC***

*Changes in MFI of cell surface markers CD80, CCR7, CCR5 and HLA-DR on monocyte-derived immature DC following culture with supernatants taken from the 48 hour co-culture of Vδ2^+^ γδ T cells with Daudi tumour cells (containing 11-52ng/ml granulysin)), as determined by flow cytometry. Treatment of cells with supernatant taken from the culture of Daudi cells alone was included as a negative control. Data shown is the average taken from 3 individual donors. Differences between groups were assessed by two-way ANOVA comparing negative controls (pre-maturation and medium alone) with all other groups. *=p<0.05. **=p<0.01.*


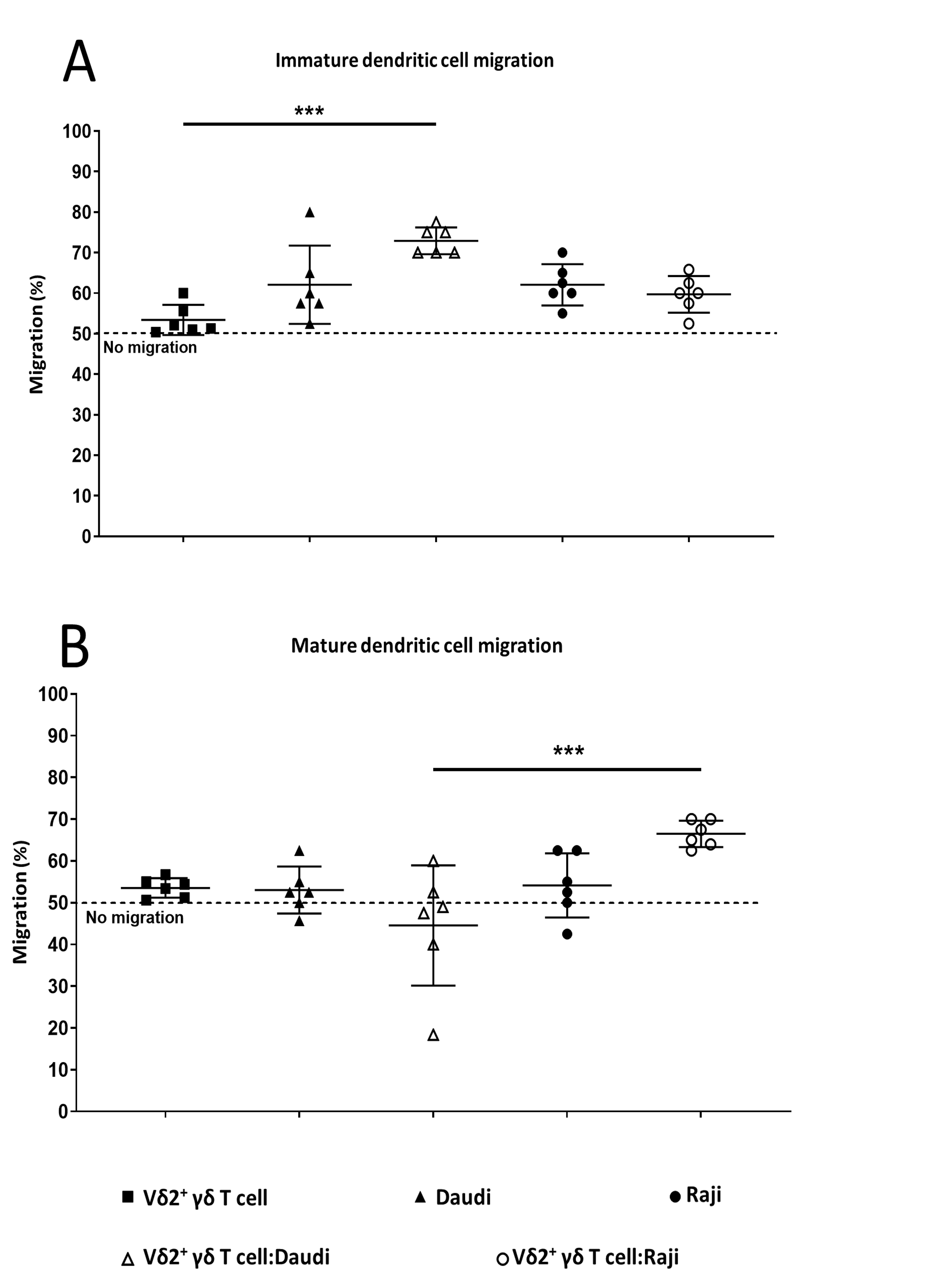


***Figure S5: Granulysin-containing supernatants cause differential migration of immature and mature DC***

*A) The migration of immature DC in response to* *supernatants taken from cultures of Daudi or Raji tumour cell lines alone, supernatants taken from cultures of Vδ2^+^ γδ T cells alone, or supernatants taken from the 48 hour co-cultures of Vδ2^+^ γδ T cells with Daudi or Raji tumour cell lines, as determined by Ibidi μ-migration assays. B*) *The migration of mature DC in response to supernatants taken from cultures of Daudi or Raji tumour cell lines alone, supernatants taken from cultures of Vδ2^+^ γδ T cells alone, or supernatants taken from the 48 hour co-cultures of Vδ2+ γδ T cells with Daudi or Raji tumour cell lines, as determined by Ibidi μ-migration assays. Data shown is from 6 independent experiments using immature and LPS-matured DC differentiated from the monocytes of 6 individual donors, with error bars (SD). Differences between groups were assessed using one-way ANOVA. ***=p<0.001*
